# Alkaline Salt Inhibits Seed Germination and Seedling Growth of Canola More Than Neutral Salt

**DOI:** 10.3389/fpls.2022.814755

**Published:** 2022-01-27

**Authors:** Weichao Wang, Fenghua Zhang, Lupeng Sun, Lei Yang, Yang Yang, Yajuan Wang, Kadambot H. M. Siddique, Jiayin Pang

**Affiliations:** ^1^The Key Laboratory of Oasis Eco-Agriculture, Xinjiang Production and Construction Crops, Shihezi University, Xinjiang, China; ^2^The UWA Institute of Agriculture and School of Agriculture and Environment, The University of Western Australia, Perth, WA, Australia

**Keywords:** salt stress, hydroponic, pH buffer systems, ion absorption and transportation, canola (*Brassica napus* L.)

## Abstract

Salinity is a major constraint to crop growth and productivity, limiting sustainable agriculture production. Planting canola (*Brassica napus* L.) variety with salinity-alkalinity tolerance as a green manure on the large area of salinity-affected land in Xinjiang could alleviate feed shortage. To investigate the differential effects of neutral and alkaline salt stress on seed germination and seedling growth of canola, we used two salts at varying concentrations, i.e., NaCl (neutral salt at 100, 150, and 200 mM) and Na_2_CO_3_ (alkaline salt at 20, 30, and 40 mM). To further explore the effects of Na^+^ and pH on seed germination, we included combined of NaCl (0, 100, 150, and 200 mM) and pH (7.1, 8.0, 9.0, 10.0, and 11.0). Shoot growth was promoted by low concentrations of NaCl and Na_2_CO_3_ but inhibited at high salt concentrations. Given the same Na^+^ concentration, Na_2_CO_3_ inhibited seed germination and seedling growth more than NaCl. The results showed that the main factor affecting seed germination and seedling growth is not pH alone, but the interaction between pH and salt ions. Under NaCl stress, canola increased the absorption of K^+^, Ca^2+^, and Mg^2+^ in roots and K^+^ in leaves. However, under Na_2_CO_3_ stress, canola maintained a high K^+^ concentration and K^+^/Na^+^ ratio in leaves and increased Ca^2+^ and Mg^2+^ in roots. Our study showed that alkaline salts inhibit canola seed germination and seedling growth more significantly than neutral salts and salt species, salt concentration, and pH significantly affected on seed germination and seedling growth. However, pH affected seed germination and seedling growth mainly through an interaction with salt ions.

## Introduction

Soil salinization is a major constraint affecting crop growth and productivity, especially in arid and semi-arid regions (Parihar et al., 2015). On a global scale, more than 800 million ha of land, i.e., nearly 20% of the total arable land area and > 7% of the total land area, are affected by salinity (Shabala, 2013; Hakim et al., 2014). Xinjiang has the largest and most widely distributed area of salinity-affected soils in China (Yang et al., 2016; Zhao et al., 2019), with 31% of cultivated land affected by varying degrees of salinization (Wang and Cheng, 2000).

Soil salinization and alkalization frequently co-occur in soil, and the conditions in natural salt/alkaline soils are very complex. Studies have shown that salt stress is caused by neutral salts, and alkali stress is induced by alkaline salts (Lin et al., 2016). Neutral salts, such as NaCl and Na_2_SO_4_, and alkaline salts, such as NaHCO_3_ and Na_2_CO_3_, are the primary ion sources found in saline soils; Na^+^, K^+^, Ca^2+^, and Mg^2+^ are the main cations and C1^–^, NO_3_^–^, HCO_3_^–^, CO_3_^2–^, and SO_4_^2–^ are the main anions. Indeed, studies have confirmed that alkaline salts damage plants more than neutral salts (Guo et al., 2015; Wang et al., 2017; Zhang et al., 2020; Lu et al., 2021). Therefore, comparing the differential response of plants to neutral and alkaline salt stress is important for improving the utilization of saline-alkaline soils for agricultural production (Wang et al., 2015).

Excessive salinity can have various adverse effects on plant, including ion toxicity, osmotic stress caused by Na^+^ and Cl^–^, accumulating, and oxidative stress due to the over-production of reactive oxygen species (Lu et al., 2017; Causin et al., 2020; Saberi Riseh et al., 2021). Salinity causes intracellular ion imbalance and decreases K^+^, Ca^2+^, and Mg^2+^ concentrations in plant (Nieves-Cordones et al., 2016; Assaha et al., 2017; Manishankar et al., 2018; Isayenkov and Maathuis, 2019; Arif et al., 2020; Shahzad et al., 2021). Intracellular K^+^ and Na^+^ homeostasis is essential for cell metabolism, with the K^+^/Na^+^ ratio determining plant enzyme activation and osmotic adjustment (Assaha et al., 2017; Pivovarov et al., 2019). Ca^2+^ is an essential component of the middle lamella and cell walls, which can alleviate Na^+^ toxicity and regulate ion absorption and transport (Amor et al., 2010; Feng et al., 2016). Therefore, preventing excessive Na^+^ transport to shoots and maintaining high cytosolic ratios of K^+^/Na^+^ and Ca^2+^/Na^+^ are crucial for salt tolerance in plants. Canola is an important oil crop and an ideal phytoremediation species for the agricultural development of saline soils in China. China is one of four major canola production areas in the world. In 2017, the planting area reached 6.65 million ha (19% of global area), with an annual yield of 13.3 million tons (17% of global production) (Food and Agricultre Organization [FAO], 2017). In general, canola is sensitive to salinity stress (Bandehagh et al., 2011; Shokri-Gharelo and Noparvar, 2018), with seed germination and seedling growth stage as the most critical and sensitive periods for most plant species subjected to salinity (Guo et al., 2017). In Xinjiang, our research group developed a saline-alkaline tolerant canola cultivar that has been widely planted (0.4 million ha) as green manure in saline and alkaline land. Therefore, it is important to investigate the physiological mechanisms underlying its salt and alkaline tolerance.

So far, studies on salt stress in canola have been primarily focused on neutral salt (NaCl), but limited studies on alkaline stress, especially the interaction between pH and salt concentrations, and its effect on seed germination and seedling growth. In this study, we investigated the exogenous application of neutral salt (NaCl) and alkali salt (Na_2_CO_3_) on seed germination, seedling growth, and the distribution of Na^+^, K^+^, Ca^2+^, Mg^2+^, Cl^–^, and SO_4_^2–^ in canola roots, shoots, and leaves. This study aimed to: (1) evaluate the effect of salt and alkali stresses on canola seed germination and seedling growth; (2) investigate the effect of salt and alkali stresses on the distribution of major saline-alkali ions in various canola plant parts; and (3) evaluate ion absorption and transport in canola plants under different saline-alkali stress conditions.

## Materials and Methods

### Plant Materials

Canola (*Brassica napus* L.) cv. Huayouza 62 was used as the experimental material, due to its high tolerance to salinity-alkalinity (Wang, 2020). The seeds were provided by Huazhong Agricultural University, China.

### Germination Experiment

#### Stress Treatments

The experiment was conducted at the Key Laboratory of Oasis Ecology Agriculture of Xinjiang Bingtuan, Shihezi University, Xinjiang, China in June 2018. Salt treatments, either as neutral salt (NaCl) or alkaline salt (NaHCO_3_), were imposed at six levels (50, 100, 150, 200, 250, and 300 mM) with little difference in pH (Table 1). The Na_2_CO_3_ stress treatments were included at ten concentrations (10, 20, 25, 30, 40, 50, 75, 100, 125, and 150 mM) to study the effects of alkaline and neutral salt stress on seed germination and seedling growth. The electrical conductivity and pH of all salt solutions were shown in Table 1.

**TABLE 1:** pH and electrical conductivity (EC) of NaCl, NaHCO_3_, and Na_2_CO_3_ solution at the various concentrations.

Treatment	Concentration		pH	EC
	mmol L^–1^	% (w/v)		(mS cm^–1^)
Control	0	0	6.05	0
NaCl	50	0.29	6.10	4.84
	100	0.59	5.91	9.24
	150	0.88	5.72	13.63
	200	1.18	5.70	18.21
	250	1.47	5.68	–
	300	1.77	5.62	–
NaHCO_3_	50	0.42	8.57	3.92
	100	0.84	8.51	7.16
	150	1.26	8.46	10.28
	200	1.68	8.37	13.21
	250	2.10	8.34	15.76
	300	2.52	8.34	–
Na_2_CO_3_	10	0.11	10.89	2.04
	20	0.21	10.99	3.73
	25	0.27	11.05	4.51
	30	0.32	11.05	5.26
	40	0.43	11.12	6.76
	50	0.53	11.15	8.12
	75	0.80	11.18	11.53
	100	1.06	11.22	14.69
	125	1.33	11.20	17.64
	150	1.60	11.19	–

*“–” indicates that the value exceeded the maximum measurement range.*

Another experiment was undertaken, with five pH levels (7.1, 8.0, 9.0, 10.0, and 11.0) and four NaCl levels (0, 100, 150, and 200 mM) to investigate the involvement of pH under salt and alkali stress on germination and seedling growth. Various pH buffer solutions were used to prepare the NaCl solution at different concentrations (Table 2).

**TABLE 2:** Chemical composition of the buffer systems, and pH and electric conductivity (EC) of the control and NaCl solutions.

pH	Buffer system	Protocol for the preparation of buffer solutions	0 mM NaCl		100 mM NaCl		150 mM NaCl		200 mM NaCl	
			pH	EC (mS cm^–1^)	pH	EC (mS cm^–1^)	pH	EC (mS cm^–1^)	pH	EC (mS cm^–1^)
7.1	Tris-HCl buffer	50 mL 0.1 M Tris solution mixed with 45.7 mL 0.1 M HCl, and diluted to 100 mL with Milli-Q water	7.5	3.5	7.6	12.2	7.6	16.4	7.6	20.6
8.0		50 mL 0.1 M Tris solution mixed with 29.2 mL 0.1 M HCl, and diluted 100 mL with Milli-Q water	8.2	2.3	8.2	11.3	8.3	15.7	8.3	19.7
9.0		50 mL 0.1 M Tris solutions mixed with 5.7 mL 0.1 M HCl, and diluted to 100 mL with Milli-Q water	9.2	0.5	9.2	9.8	9.3	14.1	9.3	18.4
10.0	NaHCO3-NaOH buffer	50 mL 0.05 M NaHCO_3_ mixed with 10.7 mL 0.1 M NaOH, and diluted to 100 ML with Milli-Q water	10.0	2.9	9.8	11.8	9.7	16.0	9.6	20.2
11.0		50 mL 0.05 M NaHCO_3_ mixed with 22.7 mL 0.1 M NaOH, and diluted to 100 ML with Milli-Q water	10.8	4.0	10.6	12.7	10.6	16.8	10.5	20.1

### Germination Test

Canola seeds of uniform size and roundness were sterilized in 0.5% NaClO for 10 min, then rinsed with sterilized Milli-Q water for five times. In each plastic germination box (10 cm × 10 cm × 5 cm), 30 seeds were spaced evenly on top of six layers of sterilized filter paper before adding 10 mL of sterilized saline solution. Each box was then covered with a lid and sealed with a sealing film. No further solution was added for the duration of the experiment. Experiment I included individual neutral salt and alkaline salt stress treatments (see Table 1), and Experiment II combined different pH levels for each NaCl treatment (see Table 2). There were three replicates per treatment, giving a total of 69 boxes in Experiment I and 60 boxes in Experiment II. The germination test was undertaken for 7 days in an artificial climate incubator set at 25°C/20°C (day/night), with relative humidity of 50–55% and daily photosynthetic photon flux density of 300 μmol photons m^–2^ s^–1^. The number of germinated seeds (when germ length reached seed length) was recorded daily. The germination energy was the germination rate recorded on the third day. After 7 days of germination, 10 seedlings with representative growth were randomly selected from each box to determine shoot length, root length, shoot fresh weight, and fresh root weight.

### Hydroponics Experiment

Based on the germination tests under salt-alkali stress, the suitable saline-alkali species (NaCl and Na_2_CO_3_) and concentration (100, 150, and 200 mM NaCl and 20, 30, and 40 Na_2_CO_3_) were selected for a hydroponics experiment (Experiment II). For germination, one seed was sown in each hole in plastic germinating trays (72 holes per tray) containing vermiculite in an artificial climate chamber with temperature set at 25°C and 14 h photoperiod. After 7 days, six seedlings in uniform size were randomly selected from the germination tray, and then transplanted into a pot (upper diameter 13.5 cm, bottom diameter 10.5 cm, and height 12.5 cm) filled with vermiculite and watered with 100 mL of 1/4 strength Hoagland nutrient solution every 2 days. Thirty-five days after growing in vermiculite when the seedlings had three true leaves, the seedlings were transferred to hydroponics with 1/4 strength Hoagland solution (Hothem et al., 2003) that was replaced every second day. Hoagland’s solution contained (mg l^–1^): MgSO_4_⋅7H_2_O 493; Ca(NO_3_)_2_⋅4H_2_O 1180; KH_2_PO_4_ 136; KNO_3_ 505; H_3_BO_3_ 2.86; MnCl_2_⋅4H_2_O 1.82; ZnSO_4_⋅7H_2_O 0.22; CuSO_4_⋅5H_2_O 0.09; MoO_3_ 0.01; Fe-DTPA 50; and had a pH of 6.5. After 14 days in hydroponics, when the seedlings had five true leaves, the nutrient solution was changed to 1/2 strength. Three days later, the following treatments were implemented: control (1/2 strength Hoagland nutrient solution), neutral stress treatments at 100 mM (low), 150 mM (moderate), and 200 mM (high) NaCl, and alkali stress treatments with at 20 mM (low), 30 mM (moderate), and 40 mM (high) Na_2_CO_3_. After 3 days, the seedlings, especially the roots, were washed in Milli-Q water to remove the residual vermiculite in the roots, and the roots, stems, and leaves were separated and harvested.

### Determination of Na^+^, K^+^, Ca^2+^, Mg^2+^, Cl^–^, and SO_4_^2–^ Concentrations

Root, stem, and leaf samples from the hydroponic experiment were oven-dried at 105°C for 30 min and then 80°C for 48 h. Approximately, 0.01 g of dried plant samples were pulverized in a crucible and then combusted in a muffle furnace (Carbolite CWF Laboratory Chamber Furnaces, CARBOLITE CWF 1300, England) for 8 h at 550°C. After cooling to room temperature, 10 mL of Milli-Q water was added to each crucible to dissolve the ash. After stirring, the solution was transferred to a 100 mL volumetric flask, and Milli-Q water was added to volume. The samples were analyzed to determine Na^+^, K^+^, Ca^2+^, Mg^2+^, Cl^–^, and SO_4_^2–^ concentrations using ion chromatography (Thermo SCIENTIFIC Ion Chromatography System DIONEX ICS-1100, Waltham, MA, United States; Cation Exchange Column: Thermo Dionex™ IonPac™ CS12A 4 mm × 250 mm and CG12A 4 mm × 50 mm; Anion Exchange Column: Thermo Dionex™ IonPac™ AS19 4 mm × 250 mm and AS19 4 mm × 50 mm).

### Statistics

The data were subjected to one- or two-way analysis of variance (ANOVA), and the least significant difference (LSD) test at *P* = 0.05 was used to determine differences among treatments using the SPSS 20.0 statistics package (SPSS, Chicago, IL, United States). The data in the Tables and Figures are expressed as mean ± standard error (*n* = 3).

## Results

### Seed Germination Rate and Seedling Growth Index

**FIGURE 1:**
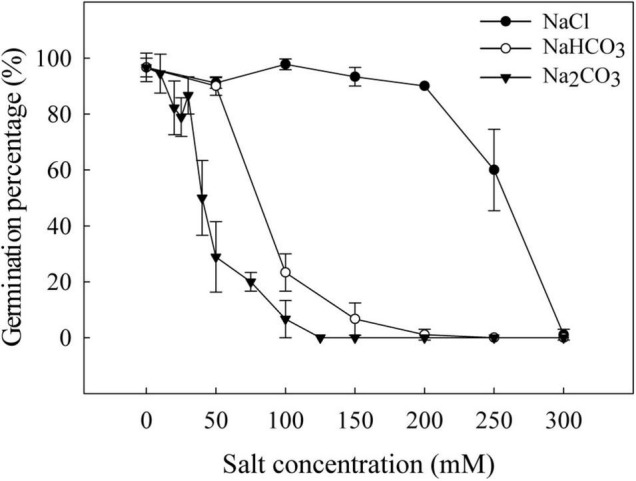
Germination percentage of canola seeds under varying levels of saline-alkali stress.

The germination percentage was > 90% from 0 mM to 200 mM NaCl (Figure 1), declining to 60% at 250 mM NaCl and almost 0% at 300 mM. The germination percentage was 90% at 50 mM NaHCO_3_, declining to 23% at 100 mM, 7% at 150 mM, and 1% at 200 mM NaHCO_3_. At 10–30 mM Na_2_CO_3_, the germination percentage was > 70%, declining to 29% at 50 mM, 7% at 100 mM, and almost 0% at 125–150 mM Na_2_CO_3_.

**FIGURE 2:**
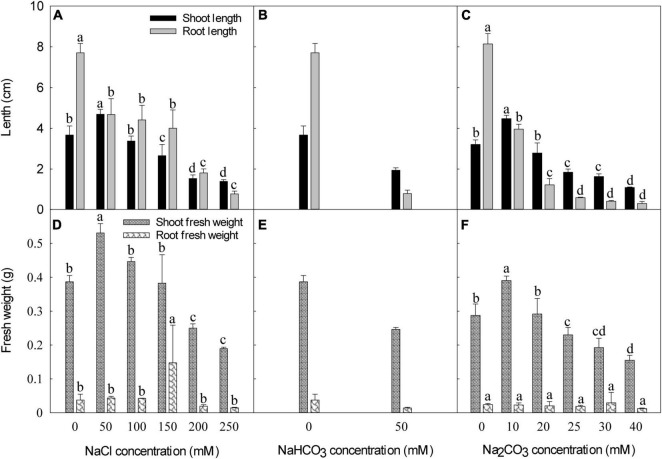
Effect of different NaCl, NaHCO_3_, and Na_2_CO_3_ concentrations on shoot and root length **(A–C)**, and shoot and root fresh weight **(D–F)** of canola seedlings 7 days after germination. Different lowercase letters indicate the significant differences between salt concentrations within the same growth parameter, according to the least significant difference (LSD) test (*P* < 0.05).

Compared with the control, shoot length and shoot fresh weight increased by 28% and 37% at 50 mM NaCl, did not significantly differ at 100 mM and 150 mM NaCl, and declined by 58% and 35% at 200 mM NaCl and 62% and 51% at 250 mM NaCl, respectively (Figures 2A,D). NaCl stress significantly decreased root length compared to the control, by 39–90% from 50 mM to 250 mM NaCl. Compared with the control, root fresh weight increased by 17% and 13% at 50 and 100 mM NaCl, respectively, and decreased by 17, 48, and 61% at 150, 200, and 250 mM NaCl, respectively. At 50 mM NaHCO_3_, shoot length, root length, shoot fresh weight, and root fresh weight declined by 47, 90, 36, and 62%, respectively, than the control (Figures 2B,E).

Low Na_2_CO_3_ (10 mM) increased shoot length and shoot fresh weight by 40 and 36% compared with the control; higher Na_2_CO_3_ concentrations decreased shoot length and shoot fresh weight. At ≥ 25 mM Na_2_CO_3_, shoot length and shoot fresh weight decreased relative to the control (Figures 2C,F). At 10, 20, 25, 30, and 40 mM Na_2_CO_3_, root length decreased by 51–96%, relative to the control.

Under the same Na^+^ concentration, the alkaline salts (Na_2_CO_3_, NaHCO_3_) had stronger inhibitory effects on seed germination and seedling growth than the neutral salt (NaCl).

### Interactive Effect of pH and Salinity on Seed Germination and Seedling Growth of Canola

**FIGURE 3:**
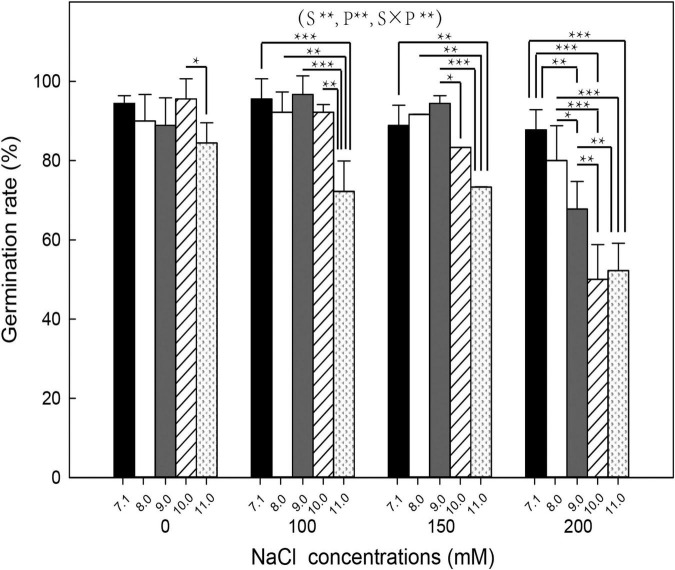
Germination percentage of canola seeds (day 7) under combined NaCl (0, 100, 150, and 200 mM) and pH level (7.1, 8.0, 9.0, 10.0, and 11.0). S is NaCl, P is pH, and S × P is their interaction, *, **, and *** indicate significance at *P* = 0.05, 0.01, and 0.001, respectively, according to the least significant difference (LSD) test for simple effects analysis of two-way ANOVA interactions (*P* < 0.05).

**FIGURE 4:**
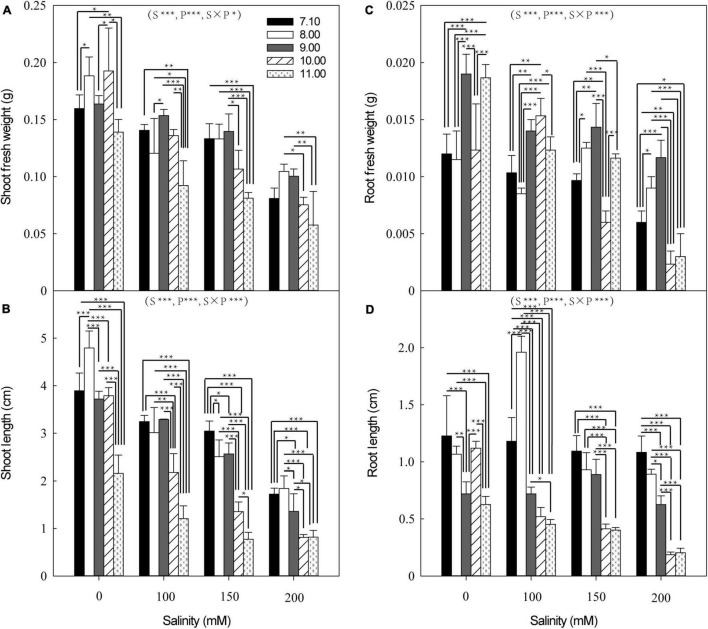
Interactive effects of NaCl and pH on shoot fresh weight **(A)**, root fresh weight **(B)**, shoot length **(C)**, and root length **(D)** of canola seedling 7 days of germination. S is salinity, P is pH, and S × P is their interaction, *, **, and *** indicate significance at *P* = 0.05, 0.01, and 0.001, respectively, according to the least significant difference (LSD) test for simple effects analysis of two-way ANOVA interactions (*P* < 0.05).

The two-way ANOVA results showed that seed germination percentage and seedling growth were affected by NaCl, pH, and their interactions (Figures 3, 4). Germination percentage decreased with increasing salinity and alkalinity (pH) (Figure 3). At pH 7.1 and 8.0, the germination percentage of canola seeds at 100, 150, and 200 mM NaCl did not significantly differ from 0 mM NaCl. At pH 9, 10, and 11, germination percentage at 100 and 150 mM NaCl did not significantly differ from 0 mM NaCl, but decreased significantly at 200 mM NaCl, with reductions of 24, 48, and 38% at pH 9.0, 10.0, and 11.0, respectively.

Shoot fresh weight (SFW, Figure 4A), shoot length (SL, Figure 4B), root fresh weight (RFW, Figure 4C), and root length (RL, Figure 4D) of canola seedlings decreased with increasing NaCl concentration at the same pH. At pH ≤ 9.0, SFW at 100 and 150 mM NaCl did not significantly differ from 0 mM NaCl, but at 200 mM NaCl, SFW decreased by 49, 44, and 39% at pH 7.1, 8.0, and 9.0, respectively (Figure 4A). At pH ≥ 10.0, SFW decreased by 29–61% with increasing salinity (100–200 mM NaCl), relative to 0 mM NaCl. At pH 7.1, SL at 100 and 150 mM NaCl did not significantly differ from 0 mM NaCl (Figure 4B), but decreased by 56% at 200 mM NaCl. At pH ≥ 8.0, SL decreased with increasing salinity (100–200 mM NaCl); more specifically, by 37–62% at pH 8.0, 12–63% at pH 9.0, 42–79% at pH 10.0, and 44–62% at pH 11.0, compared with 0 mM NaCl. At pH ≤ 9.0, RFW did not significantly differ between NaCl concentrations (Figure 4C). At pH 10.0, RFW increased at 100 mM NaCl, but decreased by 51 and 81% at 150 and 200 mM NaCl, respectively, compared with 0 mM NaCl. At pH 11.0, RFW decreased by 34, 38, and 84% at 100, 150, and 200 mM NaCl, respectively, compared with 0 mM NaCl. At pH ≤ 9.0, RL did not significantly differ between NaCl concentrations, except for pH 8.0 at 100 mM NaCl when it was greater than 0 mM NaCl (Figure 4D). At pH ≥ 10.0, RL decreased with increasing salinity, by 54, 63, and 83% at pH 10.0 and 28, 36, and 68% at pH 11.0 at 100, 150, and 200 mM NaCl, respectively, compared with 0 mM NaCl.

### Ion Changes in Canola Seedlings Under Saline-Alkali Stress

#### Na^+^ Concentration

**FIGURE 5:**
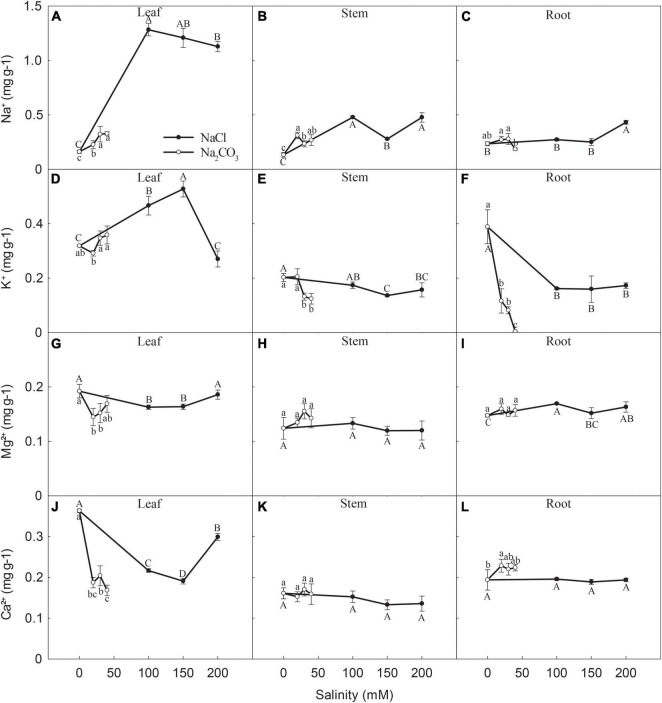
Changes in the concentrations of **(A–C)** Na^+^, **(D–F)** K^+^, **(G–I)** Mg^2+^, and **(J–L)** Ca^2+^ in the leaves, stems, and roots of canola seedlings after 7 days of NaCl and Na_2_CO_3_ stress. Different uppercase and lowercase letters indicate significant differences among NaCl and Na_2_CO_3_ concentrations, respectively, according to a least significant difference (LSD) test (*P* < 0.05).

Leaf [Na^+^] significantly increased when NaCl increased from 0 to 100 mM (696%) and 150 mM (650%), followed by a slight but significant decrease at 200 mM (599%) (*P* < 0.05), compared with 0 mM NaCl (Figure 5A). Leaf [Na^+^] increased by 41, 100, and 104% at 20, 30, and 40 mM Na_2_CO_3_, respectively, compared with 0 mM Na_2_CO_3_ (Figure 5A). Stem [Na^+^] increased by 261, 111, and 261% at 100, 150, and 200 mM NaCl and 136, 77, and 103% at 20, 30, and 40 mM Na_2_CO_3_, respectively, compared with control (Figure 5B). Root [Na^+^] remained relatively constant at 0, 100, and 150 mM NaCl, but significantly increased at 200 mM NaCl (Figure 5C). Root [Na^+^] at all Na_2_CO_3_ concentrations did not significantly differ from the control but was significantly lower at 40 mM than at 20 mM and 30 mM (Figure 5C).

#### K^+^ Concentration

Leaf [K^+^] significantly increased at 100 and 150 mM NaCl, relative to the control, but significantly decreased to close to the control at 200 mM NaCl (Figure 5D). Leaf [K^+^] in the Na_2_CO_3_ treatments did not significantly differ from the control but was significantly higher at 30 and 40 mM Na_2_CO_3_ than at 20 mM Na_2_CO_3_ (Figure 5D). Stem [K^+^] significantly decreased at 150 and 200 mM NaCl and 30 and 40 mM Na_2_CO_3_ relative to the control (Figure 5E). Root [K^+^] increased by 58, 59, and 56% at 100, 150, and 200 mM NaCl, respectively, compared to the control, with no significant differences between the three NaCl concentrations (Figure 5F). Root [K^+^] decreased by 70, 79, and 99% at 20, 30, and 40 mM Na_2_CO_3_ stress, respectively relative to the control.

#### Mg^2+^ Concentration

Leaf [Mg^2+^] significantly declined at 100 and 150 mM NaCl, relative to the control, but increased to a similar value to the control at 200 mM NaCl (Figure 5G). Leaf [Mg^2+^] significantly decreased at 20 and 30 mM Na_2_CO_3_, relative to the control, but did not significantly differ between 20, 30, and 40 mM Na_2_CO_3_ (Figure 5G). Stem [Mg^2+^] did not significantly differ among different concentrations when treated with either NaCl or Na_2_CO_3_ (Figure 5H). Root [Mg^2+^] at 100 and 200 mM NaCl increased slightly (*P* < 0.05), compared with the control, but did not significantly differ between Na_2_CO_3_ concentrations (Figure 5I).

#### Ca^2+^ Concentration

Leaf [Ca^2+^] decreased significantly at 20, 30, and 40 mM Na_2_CO_3_, relative to the control (Figure 5J). Leaf [Ca^2+^] also decreased significantly at 100 and 150 mM NaCl, compared with the control, but increased sharply at 200 mM (Figure 5J). Similar to stem [Mg^2+^], stem [Ca^2+^] varied little when treated with NaCl or Na_2_CO_3_ (Figure 5K). Root [Ca^2+^] increased significantly at 20, 30, and 40 mM Na_2_CO_3_, relative to the control, while NaCl had little effect on root [Ca^2+^] (Figure 5L).

#### Cl^–^ Concentration

**FIGURE 6:**
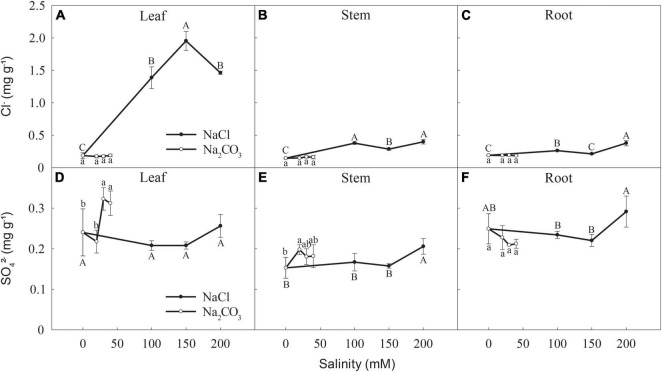
Changes in the concentrations of **(A–C)** Cl^–^ and **(D–F)** SO_4_^2–^ in the leaves, stems, and roots of canola seedlings after 7 days of NaCl and Na_2_CO_3_ stress. Different uppercase and lowercase letters indicate significant difference among NaCl and Na_2_CO_3_ concentrations, respectively, according to a least significant difference (LSD) test (*P* < 0.05).

NaCl significantly increased [Cl^–^] in leaves (Figure 6A), stems (Figure 6B), and roots (Figure 6C), relative to the control. Leaf [Cl^–^] had similar values at 100 and 200 mM NaCl, and the highest value at 150 mM. Stem [Cl^–^] was highest at 100 and 200 mM NaCl, followed by 150 mM. Root [Cl^–^] had the highest value at 200 mM, followed by 100 mM, with no significant difference between 150 mM and the control (Figure 6C). The Na_2_CO_3_ treatments had little effect on [Cl^–^] in leaves, stems, or roots (Figures 6A–C).

#### SO_4_^2–^ Concentration

Leaf [SO_4_^2–^] at 30 and 40 mM Na_2_CO_3_ significantly increased relative to the control and 20 mM Na_2_CO_3_, but there were no significant effects of the NaCl treatments (Figure 6D). Stem [SO_4_^2–^] significantly increased at 20 mM Na_2_CO_3_, relative to the control, but did not significantly differ at 30 and 40 mM Na_2_CO_3_ (Figure 6E). Stem and root [SO_4_^2–^] at 200 mM NaCl significantly increased relative to the control but did not significantly differ at 100 mM and 150 mM NaCl (Figures 6E,F). The Na_2_CO_3_ treatments did not affect root [SO_4_^2–^] (Figure 6F).

#### K^+^/Na^+^, Ca^2+^/Na^+^, and Mg^2+^/Na^+^ Ratios in Different Tissues

The K^+^/Na^+^ ratio in roots, stems, and leaves of canola seedlings decreased significantly with increasing Na_2_CO_3_ concentration, relative to the control (Table 3). Among the different tissues, the K^+^/Na^+^ ratio under Na_2_CO_3_ followed the order of leaves > stems > roots (*P* < 0.05). The K^+^/Na^+^ ratio decreased significantly with increasing NaCl concentration, compared with the control; and it was higher in the roots than that in the leaves and stems (*P* < 0.05).

**TABLE 3:** Changes in the ratios of K^+^/Na^+^, Ca^2+^/Na^+^, and Mg^2+^/Na^+^ ratios in different tissues of canola seedlings after 7 days of NaCl and Na_2_CO_3_ stress.

Treatment	Concentration (mM)	K^+^/Na^+^			Ca^2+^/Na^+^			Mg^2+^/Na^+^		
		Root	Stem	Leaf	Root	Stem	Leaf	Root	Stem	Leaf
Control	0	1.67 ± 0.39aA	1.55 ± 0.16aA	1.99 ± 0.18aA	0.82 ± 0.08aC	1.23 ± 0.15aB	2.26 ± 0.18aA	0.63 ± 0.04aC	0.94 ± 0.02aB	1.19 ± 0.03aA
NaCl	100	0.59 ± 0.02bA	0.36 ± 0.03bB	0.36 ± 0.01bcB	0.72 ± 0.02aA	0.32 ± 0.04cB	0.17 ± 0.01bC	0.62 ± 0.03aA	0.28 ± 0.03cB	0.13 ± 0.01cC
	150	0.64 ± 0.17bA	0.49 ± 0.03bA	0.44 ± 0.06bA	0.76 ± 0.12aA	0.48 ± 0.05bB	0.16 ± 0.01bC	0.61 ± 0.07aA	0.43 ± 0.04bB	0.14 ± 0.01bcC
	200	0.40 ± 0.01bA	0.33 ± 0.03bB	0.24 ± 0.04cC	0.45 ± 0.01bA	0.28 ± 0.02cB	0.27 ± 0.02bB	0.38 ± 0.04bA	0.25 ± 0.02cB	0.17 ± 0.01bC
Control	0	1.67 ± 0.39aA	1.55 ± 0.16aA	1.99 ± 0.18aA	0.82 ± 0.08bC	1.23 ± 0.15aB	2.26 ± 0.18aA	0.63 ± 0.04bC	0.94 ± 0.02aB	1.19 ± 0.03aA
Na_2_CO_3_	20	0.43 ± 0.16bB	0.66 ± 0.05bB	1.31 ± 0.18bA	0.85 ± 0.12bA	0.49 ± 0.04cB	0.84 ± 0.17bA	0.59 ± 0.03bA	0.43 ± 0.03cB	0.65 ± 0.10bA
	30	0.31 ± 0.11bB	0.57 ± 0.08bB	1.11 ± 0.23bA	0.81 ± 0.19bA	0.73 ± 0.07bA	0.66 ± 0.18bcA	0.55 ± 0.11bA	0.67 ± 0.08bA	0.49 ± 0.13bA
	40	0.03 ± 0.00bC	0.47 ± 0.12bB	1.09 ± 0.05bA	1.21 ± 0.09aA	0.60 ± 0.11bcB	0.51 ± 0.06cB	0.85 ± 0.03aA	0.54 ± 0.10bcB	0.51 ± 0.06bB

*Different lowercase letters in the same column indicate significant differences among NaCl or Na_2_CO_3_ concentrations, and different uppercase letters in the same row indicate significant differences among plant tissues within the same treatment, according to a least significant difference (LSD) test (P < 0.05).*

Both NaCl and Na_2_CO_3_ stress significantly decreased the Ca^2+^/Na^+^ and Mg^2+^/Na^+^ ratios in stems and leaves; however, those ratios in the roots at 100 and 150 mM NaCl and 20 and 30 mM Na_2_CO_3_ were similar to the control. Root Ca^2+^/Na^+^ and Mg^2+^/Na^+^ ratios at 200 mM NaCl significantly decreased relative to the controls, but they significantly increased at 40 mM Na_2_CO_3_. Roots had significantly higher Ca^2+^/Na^+^ and Mg^2+^/Na^+^ ratios than stems and leaves in the same NaCl treatment. The Ca^2+^/Na^+^ and Mg^2+^/Na^+^ ratios did not significantly differ between roots and leaves at 20 and 30 mM, but were higher in roots than stems and leaves at 40 mM.

## Discussion

### Comparison of Different Types of Saline-Alkali Stress on Seed Germination and Seedling Growth

Low salinity (50 mM NaCl and 10 mM Na_2_CO_3_) promoted shoot growth of canola seedlings, but high salt stress significantly inhibited seed germination (Figure 1) and plant root growth (Figure 2). At the same Na^+^ concentration, alkaline salt stress (Na_2_CO_3_ or NaHCO_3_) inhibited seed germination and seedling growth more than neutral salt stress (NaCl), which may be due to a pH effect for alkaline salt in addition to the ion factor caused by neutral stress (Wang et al., 2011, 2017). At the same Na^+^ concentration, Na_2_CO_3_ had higher pH and EC than NaHCO_3_, but the germination percentage was higher under Na_2_CO_3_ than under NaHCO_3_, suggesting that factors other than pH (e.g., ion type and ion concentration) affect germination and seedling growth in canola.

### Interactive Effects of pH and Salinity on Seed Germination and Seedling Growth

Salinity, pH, and their interaction significantly affected seed germination and seedling growth of canola. At 100 mM NaCl, the germination rate reduced significantly at high pH (≥11.00), while at 200 mM NaCl, the germination rate was significantly reduced at pH ≥ 9.0, these results demonstrate the interaction of pH and salinity. However, Zhao et al. (2014) have reported that non-significant interactions between Na^+^ concentration and pH on germination rate. Some studies have also shown significantly higher interactive effects of salinity and high pH on plants than salinity or pH alone (Li et al., 2010; Hu et al., 2018; Lin et al., 2018), but this was demonstrated more in this study by introducing a pH buffer system that excluded other factors. High pH reduced the average germination time and increased the germination rate of sorghum even under low Na^+^ concentration (Zhao et al., 2014). Compared with the individual alkali stress (Na_2_CO_3_), canola seedlings treated with 100 mM NaCl at pH 11.0 had a much higher germination percentage than those treated with 50 mM Na_2_CO_3_ at pH 11.15, despite both treatments having the same Na^+^ concentration and pH value. These results suggest that species of cations and anions affect canola germination and growth, in addition to pH and salt concentration.

### Ion Accumulation

Under saline-alkali stress, plants can enhance selective ion absorption by roots and regulate their distribution within the plant to achieve a stable ion status, as an underlying mechanism of salinity tolerance (Ben-Amor et al., 2010; Golldack et al., 2014; Cao et al., 2020). We showed that root, stem, and leaf Na^+^ concentrations in canola seedlings increased under NaCl and Na_2_CO_3_ stress while root and stem K^+^ concentrations decreased due to competition between Na^+^ and K^+^ absorption. Studies have shown that salinity reduced K^+^ and Ca^2+^ absorption in plants (Liu et al., 2014; Assaha et al., 2017; Isayenkov and Maathuis, 2019; Arif et al., 2020). Excess Na^+^ influx into the cytoplasm under salt stress depolarizes the membrane potential, which activates K^+^ outward rectifier channels, resulting in K^+^ efflux from root and leaf cells (Demidchik et al., 2014; Assaha et al., 2017). Regulating K^+^ homeostasis (inhibiting K^+^ efflux) and maintaining a high K^+^/Na^+^ ratio are critical for salinity tolerance in plants (Janicka-Russak and Kabała, 2015; Falhof et al., 2016; Zhang et al., 2017; Yang and Guo, 2018). Studies have shown that the PM H-ATPases, K^+^ transporters, Na^+^/H^+^ exchangers (NHX) and the antiporter, salt overly sensitive 1 (*SOS1*) act synergistically to mitigate the effects of salt stress and low K^+^ on plant growth (Janicka-Russak and Kabała, 2015; Chakraborty et al., 2016; Falhof et al., 2016).

In this study, plants under NaCl stress had lower Na^+^ and Cl^–^ concentrations in roots than stems and leaves (Figures 5, 6), indicating that Huayouza 62 has a low capacity to retain saline ions in roots. In contrast, higher Na^+^ concentrations occurred in roots than stems and leaves in potato (*Solanum tuberosum* L.) (Queirós et al., 2009) and pepper (*Capsicum chinense* Jacq.) (Emanuel et al., 2014) under NaCl stress. Higher Na^+^ concentrations in roots generally maintain osmotic potential and prevent translocation to leaves, avoiding leaf Na^+^ accumulation (Xue et al., 2013). A study in canola seedlings showed that leaf Na^+^ distribution was highest under NaCl stress but confined primarily to the leaf edge and restricted in leaf apoplasts, protecting cells from Na^+^ stress (Gao et al., 2016). In addition, rice (*Oryza sativa* L.) and potato (*Solanum tuberosum* L.) plantlets under saline conditions accumulate Cl^–^ to neutralize the large amounts of cations and maintain stable intracellular pH (Wang et al., 2011; Gao et al., 2015).

In this study, NaCl stress inhibited Ca^2+^ and Mg^2+^ accumulation, especially in leaves, while Na_2_CO_3_ stress enhanced Ca^2+^ and Mg^2+^ accumulation in stems and roots. Increasing NaCl significantly increased the amount of Na^+^ in *Suaeda salsa* plants but decreased Ca^2+^ and Mg^2+^ concentrations (Guan et al., 2011). Other studies have shown that alkali stress significantly increased root Ca^2+^ and Mg^2+^ contents and shoot Mg^2+^ content (Guo et al., 2017; Wang et al., 2017). Ca^2+^ and Mg^2+^ can control the ionic balance of cells by regulating selective ion absorption and transport, reducing toxicity under saline-alkali stress (Ben-Amor et al., 2010; Feng et al., 2016; Manishankar et al., 2018). In addition, Ca^2+^ plays an important role in maintaining cell membrane stability and preventing membrane damage (Liu et al., 2014; Feng et al., 2016). Increased Ca^2+^ levels in the tissues of maize seedlings exposed to salt stress may have activated the salt overly sensitive (*SOS*)-Na^+^ system for exclusion and reduced plant damage caused by Na^+^ toxicity (Guo et al., 2017).

Under NaCl stress, canola roots had significantly higher K^+^/Na^+^, Ca^2+^/Na^+^ and Mg^2+^/Na^+^ than stems and leaves, and the K^+^/Na^+^ ratios in leaves and the Ca^2+^/Na^+^ and Mg^2+^/Na^+^ ratios in roots and stems decreased significantly at high NaCl concentration. Under Na_2_CO_3_ stress, canola leaves had a significantly higher K^+^/Na^+^ ratio than roots and stems, which decreased with increasing Na_2_CO_3_ concentration; in contrast, the Ca^2+^/Na^+^ and Mg^2+^/Na^+^ ratios in roots increased significantly with increasing Na_2_CO_3_ concentration. This may be due to the high pH environment outside roots, therefore reducing the number of protons in external solution, weakening the exchange activity of the Na^+^/H^+^ antiport in the root plasma membrane (Cao et al., 2020). A weakened Na^+^/H^+^ antiport reduces the exclusion of Na^+^ into the rhizosphere, enhancing plant Na^+^ accumulation. In addition, studies have shown that in dicots stems, HKT, and SOS1 mediate Na^+^ exclusion by retrieving Na^+^ from the xylem into xylem parenchyma cells, and reducing the amount of Na^+^ transported from xylem to the shoot (Assaha et al., 2015, 2017), which may also be an important process for rape to reduce Na ion content in leaves. These results indicate that maintaining a high K^+^ concentration and K^+^/Na^+^ ratio in leaves and increasing the absorption of Ca^2+^ and Mg^2+^ in roots might be important mechanisms underlying alkali tolerance in canola plants.

## Conclusion

Low salinity (50 mM NaCl and 10 mM Na_2_CO_3_) increased shoot growth in canola seedlings, while high salinity (200 mM NaCl and 40 mM Na_2_CO_3_) significantly inhibited germination and seedling growth. The alkaline salt (Na_2_CO_3_) stress restricted seed germination and seedling growth more than the neutral salt (NaCl) stress. Under alkaline salt stress, the interaction of pH and salt ions rather than pH was the most important factor affecting seed germination and seedling growth. Ion absorption and balance in canola seedlings differed under neutral and alkaline salt stress. Under neutral salt stress, salt tolerance improved in canola due to increased root K^+^, Ca^2+^, and Mg^2+^ absorption and increased leaf K^+^. Under alkaline salt stress, canola maintained a high K^+^ concentration and K^+^/Na^+^ ratio in the leaves and increased root Ca^2+^ and Mg^2+^ uptake. Therefore, ionic regulation may be an important mechanism underlying alkaline salt tolerance in canola. However, further experiments should be conducted to assess the germination of canola seeds under individual salt ion stress conditions and the enzymatic activities and cellular damages at different stages of the germination process, and to elucidate the ion regulatory mechanisms in conjunction with transporter protein activity, in order to better understand the effects of salinity stress on canola seeds during germination and the ionic mechanisms of their response.

## Data Availability Statement

The original contributions presented in the study are included in the article/supplementary material, further inquiries can be directed to the corresponding author.

## Author Contributions

WW and FZ designed the research. WW, LS, YY, and YW performed the experiments. WW, JP, and LY performed the data analysis and interpretation, and prepared the figures and tables. WW, FZ, JP, and KS wrote the manuscript. All authors read, commented on- and approved the manuscript.

## Conflict of Interest

The authors declare that the research was conducted in the absence of any commercial or financial relationships that could be construed as a potential conflict of interest.

## Publisher’s Note

All claims expressed in this article are solely those of the authors and do not necessarily represent those of their affiliated organizations, or those of the publisher, the editors and the reviewers. Any product that may be evaluated in this article, or claim that may be made by its manufacturer, is not guaranteed or endorsed by the publisher.
